# Variation of Durability and Strength Parameters of Pumice Based Mixtures

**DOI:** 10.3390/ma14133674

**Published:** 2021-07-01

**Authors:** Petr Lehner, Petr Konečný, Pratanu Ghosh

**Affiliations:** 1 Department of Structural Mechanics, Faculty of Civil Engineering, VSB-Technical University of Ostrava, Ludvíka Podéště 1875/17, 708 33 Ostrava-Poruba, Czech Republic; petr.konecny@vsb.cz; 2 Civil and Environmental Engineering Department, California State University, Fullerton, CA 92834, USA; pghosh@fullerton.edu

**Keywords:** concrete, chloride, pumice, coefficient of variation, diffusion coefficient, trend line

## Abstract

The numerical modelling of chloride penetration into concrete is very sensitive to the correct description of the input data. In the recent era, high-performance concrete (HPC), which combines Portland cement and other supplementary cementitious materials, has been gaining attraction due to their desirable material properties and durability. The presented results show the application of the modified approach for the evaluation of the suitability of the time-dependent model for the variation of the diffusion coefficient. The 26 various binary and ternary-based concrete mixtures blended with volcanic pumice pozzolan (VPP) as a major supplementary cementitious material (SCM) are compared with the reference Ordinary Portland Cement mixture. Other SCMs namely fly ash, slag, silica fume, and metakaolin were also utilized in ternary-based concrete mixtures. In-depth statistical analysis was carried out to show the variability and effects of the amount of the volcanic pumice as an SCM on the diffusion coefficient. The mean value and regression via linear approximation of the time-dependent coefficient of variation of the diffusion coefficients were used as well as the Root of Mean Squared Error approach. The presented results are suitable as the component of the input parameters for the durability-related probabilistic assessment of the reinforced concrete structures exposed to chlorides. In addition, the time-dependent ultimate limit state-related data was presented.

## 1. Introduction

In the sustainable design of the concrete structures, it is necessary to find the balance between the material side (i.e., the development of new concrete mixtures with the required properties [1,2,3]), an appropriate description of performance (i.e., testing and proper statistical evaluation [4,5,6]), and structural and modelling components (i.e., sufficiently durable and sustainable design [7,8,9,10]). All three components must be considered together and looked at from the perspective of the studied problem, financial demands, production, or a possible estimate of the reconstruction of the proposed structure. A typical example of factors significantly influencing the structural durability is the resistance of reinforced concrete structures to corrosion of reinforcement caused by external factors, such as the penetration of chloride ions that occurs when de-icing roads [11,12,13]. This problem is also typical for the reinforced concrete structures exposed to the marine environment [14,15]. The goal is to find a way to increase the durability of reinforced concrete structures with respect to the resistance to such external environmental parameters.

The sustainability factor is important as well because the production of cement emits about 8% of total CO_2_ emissions and the replacement of Portland cement by natural pozzolans significantly reduces the emission of CO_2_ [16,17]. The first point of view, i.e., the search for new materials, both with respect to durability as well as sustainability, may be related to the replacement of cement by some additional cementitious materials (SCM). Many research groups and corporations are dealing with the preparation of concrete from volcanic ash, fly ash, blast furnace slag, silica fume, metakaolin, or pumice, etc. [18,19,20,21]. These research studies have shown that the Portland cement replacement leads to improved material properties of the resulting concrete mixture including diffusive parameters that are involved in durability analysis and, therefore, these mixtures are more suitable. For example, the recent study with VPP-based concrete mixtures [18] confirmed that the electrical resistivity is time-varying as expected and therefore the diffusion coefficient is time-dependent as well. Sustainability indicators for pumice-based concrete mixtures were also examined [22], which highlighted the balance between sustainability performance. It is worth mentioning that sustainability potential indicator *k*_sb_, applied in [22], evaluated the trade-off between the performance, namely strength and durability, with the eco costs. This allowed evaluating the balance between the reliability, economic and ecological aspects that are essential for sustainable design. The second point of view, i.e., the appropriate evaluation of laboratory results and their correct statistical evaluation, is of similar importance. Since concrete is a heterogeneous material that changes its properties significantly over time, the analysis of diffusion parameter data measured in the laboratory is of great importance [23]. Such analysis was demonstrated by a study on a different set of binary and ternary high-performance concrete mixtures [4], where the entire process of in-depth statistical analysis of several binary and ternary concrete mixtures blended with various SCMs is described in a detailed manner. The study [4] focused on SCMs namely metakaolin, silica fume, fly ash Class F, fly ash Class C, and ground granulated blast furnace slag class 120. The evaluation of variation of diffusion coefficient random variation time dependence on the set of studied SCMs showed the possibilities of approximation of the time-dependent behaviour via dimensionless constant or linearly regressed coefficient of variation of the diffusion coefficient. The quality of the approximation was studied via Root Mean Squared Method (RMSE). The third important issue is the prediction of the behaviour of the time-dependent performance of deteriorated reinforced concrete structures. The probabilistic time-dependent analysis of chloride ingress or even loss of carrying capacity related to the chloride-induced corrosion was already conducted in depth e.g., in [24,25,26,27]. It is worth mentioning that the structural performance of the carrying capacity, evaluated in [24,25], is reduced by the corrosion-induced losses in steel reinforcement. However, the effect of concrete maturity level and age concrete parameters such as chloride diffusion coefficient or strength itself are not considered as a function of time [28]. Moreover, it is important to consider that the evaluated reliability level depends on the selection of a probabilistic distribution function of selected input parameters as well as the proper limit state definition [29]. Therefore, research on time-dependent variation of durability and strength parameters of pumice-based mixtures requires further attention. The presented study focuses mainly on the second part, i.e., the evaluation of laboratory experimental data in such a way that the results can be used as input parameters for optimal modelling of the probabilistic analysis of the durability of concrete structures with the probabilistic approach. Initial data from electrochemical tests of volcanic pumice pozzolan (VPP)-based concrete mixtures [18,30] are accumulated, diffusion parameters are determined, and their variance is described using standard deviation and coefficient of variation. The analysis is based on improved evaluation of RMSE that was published in [4], and evaluating in-depth statistical analysis of VPP-based binary and ternary concrete mixtures including the time-dependent strength approximation.

## 2. Research Significance

The concrete material parameters, in general, show random variability, specifically mechanical parameters, as well as diffusive parameters. In the case of evaluation of the durability of corrosion initiation of steel reinforcement, many suitable tools handling the durability of concrete structures are available [6,25,29,31,32], which can be combined with appropriate numerical probabilistic durability assessment such as e.g., [8,33,34]. However, the reliable description of random input parameters is often a problem. Therefore, the research of the variability of new VPP SCM mixture from the chloride ingress as well as including a deterministic description of time-dependent strength approximation is significant with respect to the preparation of the probabilistic numerical model. Besides the time-dependent behaviour of electrical resistivity of VPP mixtures [18], the diffusion parameter derived from the experimental data can be considered also as a time-dependent parameter. Therefore, the analysis of diffusion parameters evaluates how it changes over time in the case of individual concrete mixtures sorted into groups. The experimental data are compared with mean and linear regression approximations of variation coefficients for each pumice mixture blended with several other cementitious materials namely Class C, Class F, Slag 120, silica fume, and metakaolin. Such analysis is conducted with the modified RMSE methodology presented in [4]. The RMSE approach is modified to be relative (dimensionless) to study the quality of the time-dependent behaviour fit in an easier way. The selection of the suitable approximation is made if the difference in both methods is more than 10%.

## 3. Materials and Experimental Investigation

### 3.1. Mixture Design

Twenty-six volcanic pumice-based mixtures with a water/cementitious materials ratio of 0.44 were prepared for this comprehensive experimental study. Most of the exposed bridge decks and substructure concrete’s mix design are prepared with this typical water–cementitious materials ratio. Table 1 summarizes SCM in all the concrete mixtures.

For all cement mixtures, a 19 mm limestone coarse aggregate with a coarse aggregate factor (CAF) of 0.67 was selected, which meets ASTM C33 specifications. In addition, ASTM C33 silica sand was used as a fine aggregate. The specific gravity of coarse aggregate was 2.66 and fine aggregate 2.65. All SCMs were replaced with mass. The experiments were performed using type II-V (TII-V) cement (medium type II and type V sulphate cement mixed together) in accordance with ASTM C150 and various cementitious materials, namely, ground granulated blast furnace slag class 120 (G120S), Class F fly ash (F), Class C fly ash (C), silica fume (SF), metakaolin (M) and volcanic pumice stone (P). All experimental laboratory research was performed at California State University in Fullerton and numerical analysis was performed in the Czech Republic. California has a major problem with sulfate attacks in concrete. Therefore, it is recommended to use type II-V cement instead of Portland type I cement. The selection of different concrete mixes was based on the criteria of meeting the basic technical properties and representing a diverse range of solutions for different durability problems. The nomenclature of the mix parameters was chosen based on the weight percentage of each cementitious material, e.g., 75TII-V/20P/5SF means 75% type II-V cement, 20% volcanic pumice, and 5% silica smoke. High-range water reducers and air admixtures have been used to achieve better workability and durability. A water reducer was used in the range of 10–14 oz per 100 pounds of cementitious materials and an air filter was used in the range of 0.6 to 1.4 oz per 100 pounds of cementitious materials.

### 3.2. Slump and Air Content

**Figure 1:**
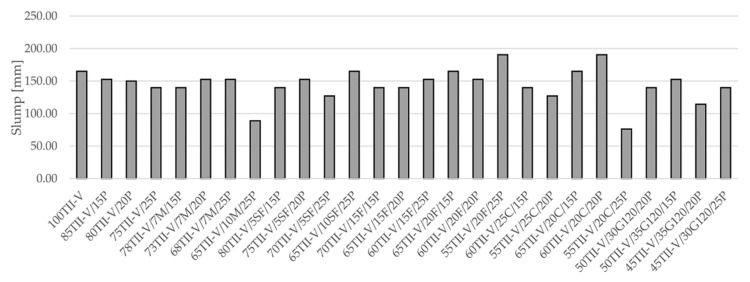
Results of the slump test for all mixtures.

**Figure 2:**
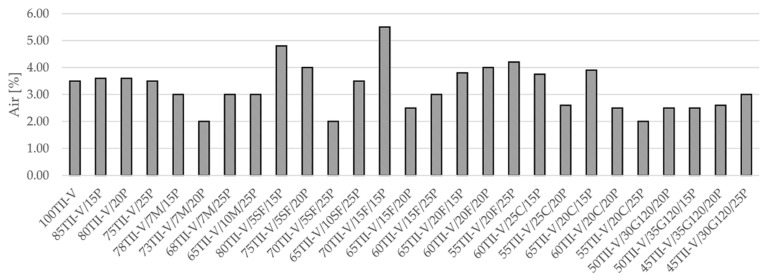
Results of the air content for all mixtures.

Selected material properties of studied mixtures, namely slump and air content, are shown in Figure 1 and Figure 2. The reference concrete with the Mix ID 100TII-V has a slump value of 165 mm, while the average slump of the VPP mixtures is 144 mm. The lowest value of slump (76 mm) has the concrete 55TII-V/20C/25P, while the highest value (190 mm) has the concrete 55TII-V/20F/25P. In general, Class F fly ash has lower water demand and it works well with pumice compared to Class C fly ash as it has more cementitious properties rather than pozzolanic properties. It is to be noted that pumice is a very fine material compared to other pozzolans, except silica fume and metakaolin. Hence, it has higher water demand compared to OPC, Class F fly ash, or slag 120. However, interaction pumice with other SCM shows variable slump values. In most pumice-based binary and ternary mixtures, a large amount of medium-range water-reducing admixtures were added to achieve a target slump of 125 mm for better workability.

The reference concrete has an air content of 3.5%, while the average slump of the VPP mixtures is 3.2%. The lowest value of air content (2%) has the concretes 73TII-V/7M/20P, 70TII-V/5SF/25P, and 55TII-V/20C/25P, while the highest value (5.5) has the concrete 70TII-V/15F/15P. It is to be noted that all mixtures were cast with air-entertaining admixture to achieve a target moderate air content of 3%. However, the target was not met in all mixtures even after two trials of mixing. Further study needs to be conducted to observe the effect of air-entraining admixture on various pozzolans.

### 3.3. Compressive Strength

**Figure 3:**
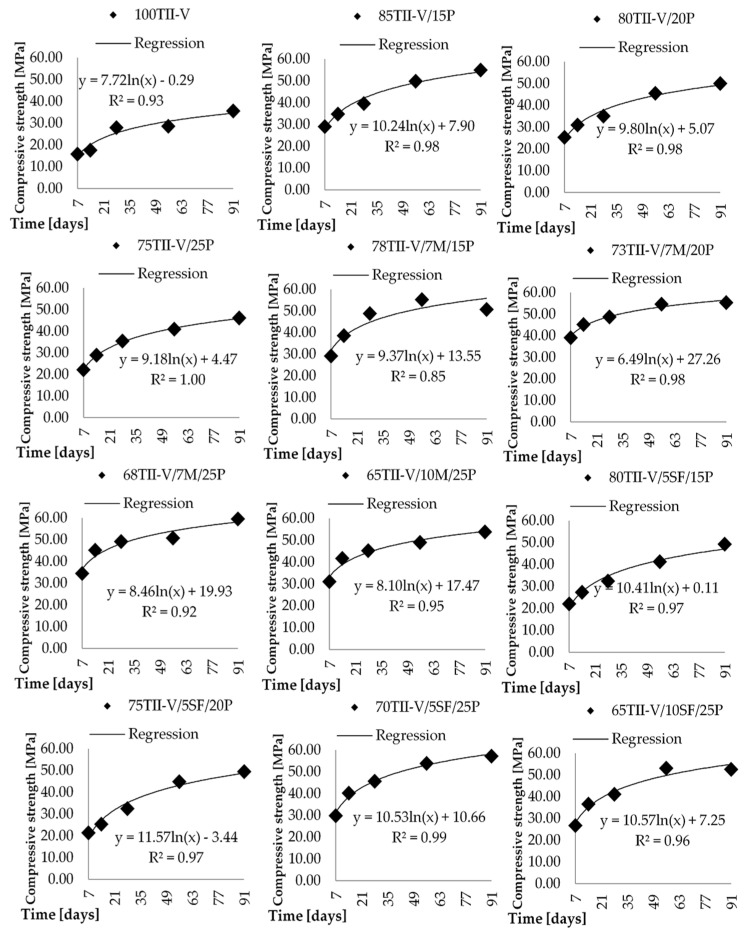
Correlation of strength versus concrete age for pumice, metakaolin, and silica fume-based mixtures.

**Figure 4:**
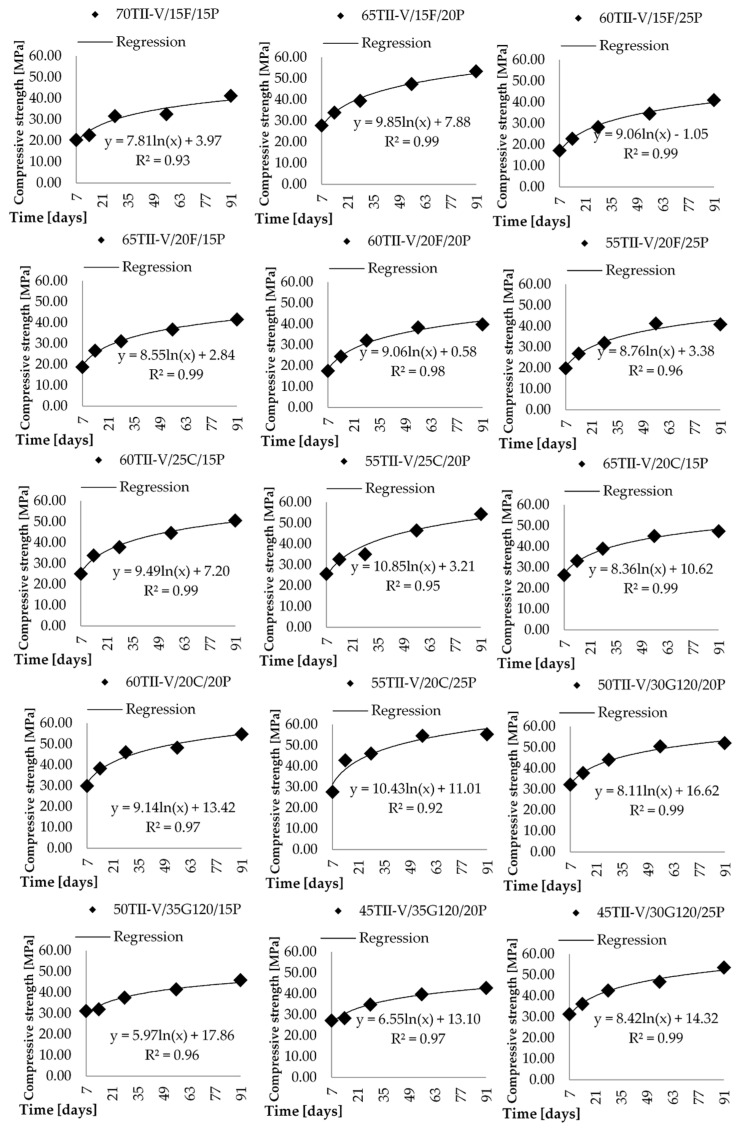
Correlation of strength versus concrete age for pumice, metakaolin, and silica fume-based mixtures.

The compressive strength obtained on cylinders (100 mm diameter, 200 mm length) is presented as a time-dependent regression function in Figure 3 and Figure 4. The cylinders were taken out from the mold after 24 ± 2 h and were placed in a limewater tank for the continuous curing process.

It can be observed that the concrete mixtures are gaining strength as the hydration and pozzolanic reaction process progress over time. Even though it is more significant in the case of concrete with volcanic pumice SCM, the rate of increase in strength was computed by comparing a ratio of 28 days strength with 56 days or 91 days strength. The reference concrete has *f*_c,56_/*f*_c,28_ = 102%, while the *f*_c,91_/*f*_c,28_ = 127%. The average value of the VPP mixtures is *f*_c,56_/*f*_c,28_ = 119%, while the *f*_c,91_/*f*_c,28_ = 129%. It can be observed that the difference between the 28th and 56th day results is significant between the OPC and the average of VPP, while the difference between the 28th and 91st day results is almost negligible.

The lowest value of *f*_c,56_/*f*_c,28_ = 103% belongs to the mixture 70TII-V/15F/15P and 68TII-V/7M/25P, while the highest value (138%) is obtained for the mixture 75TII-V/5SF/20P. The lowest value is almost the same as the reference one while the highest is significantly better.

The results of the 28th and 91st day strength ratio comparison show wide extreme distribution. The lowest value of *f*_c,91_/*f*_c,28_ = 104% belongs to the mixture 78TII-V/7M/15P, while the highest value (154%) is observed for the mixture 55TII-V/25C/20P.

### 3.4. Diffusion Coefficient Calculation

The bulk conductivity measurement at the ages of 7, 14, 28, 56, and 91 days was measured on four concrete cylindrical specimens with dimensions of diameter 100 mm and height 200 mm. Readings of electrical resistivity were obtained two times at one concrete age by placing the concrete cylinder inside the terminal connected to the electrode plates and the data logger connected to the computer, which records the bulk conductivity data (a detailed description of the measurement is provided in [15,22]).

Thus, a total of eight measurements were performed on each mixture at each time point. The bulk conductivity was converted to the resistivity, and then the diffusion coefficient was calculated using the theoretical electrochemical equation known as Nernst-Einstein [35]:

(1) $$D=\frac{RT}{Z^{2}F^{2}}\cdot \frac{t_{i}}{\gamma_{i}C_{i}\rho_{BR}}$$

where *D* is the diffusion coefficient (m^2^/s), *R* is the universal gas constant (J/K·mol), *T* is the absolute temperature (K), *Z* is the valence of ions (-), *F* is the Faradays constant (C/mol), *t*_i_ is the transport number of chloride ions (-), *γ*_I_ is the activity coefficient of chloride ions (-), *C*_i_ is the concentration of chloride ions (mol/m^3^), and *ρ*_*BR*_ is the bulk resistivity (Kohm-cm).

The description of the time-dependent diffusion coefficient model may be based on the reference diffusion coefficient. Thus, the diffusion coefficient is a time-dependent parameter [23,36]; it can be calculated by the equation:

(2) $$D_{c,\mathrm{nom},t}=D_{c,28}\cdot {\left(\frac{t_{28}}{t}\right)}^{m}$$

where *D*_c,nom,*t*_ is the nominal diffusion coefficient for a selected age (m^2^/s), *m* is the ageing factor describing the decrease of the diffusion coefficient over the period of measurement for concrete ages *t* (years) (e.g., 7, 14, 28, 56, 91, and 161 days), and *t*_ref_ (years) is the age-related to the diffusion coefficient *D*_c,ref_ at reference period e.g., 28 days. The reference parameters and the *m* factor might be computed according to [37]. The calculated ageing factor is shown in Table 2. The input parameters for the deterministic or the nominal value of the time-dependent diffusion coefficient model according to Equation (2), computed with the Least square method approximation, are in Table 2. Moreover, the 28th day compressive strength complements the durability-related data in Table 2.

From the parameters applied in Equation (1), the standardized statistical descriptors are evaluated, i.e., mean diffusion coefficient *µ* (m^2^/s) and coefficient of variation *c_v_* (-) of each concrete mixture were determined per age concrete maturity period. The coefficient of variation *c_v_* (-) is computed as standard deviation (m^2^/s) divided by mean value *µ* (m^2^/s). Both parameters are shown in Table 2.

## 4. Diffusion Coefficient Analysis

**Figure 5:**
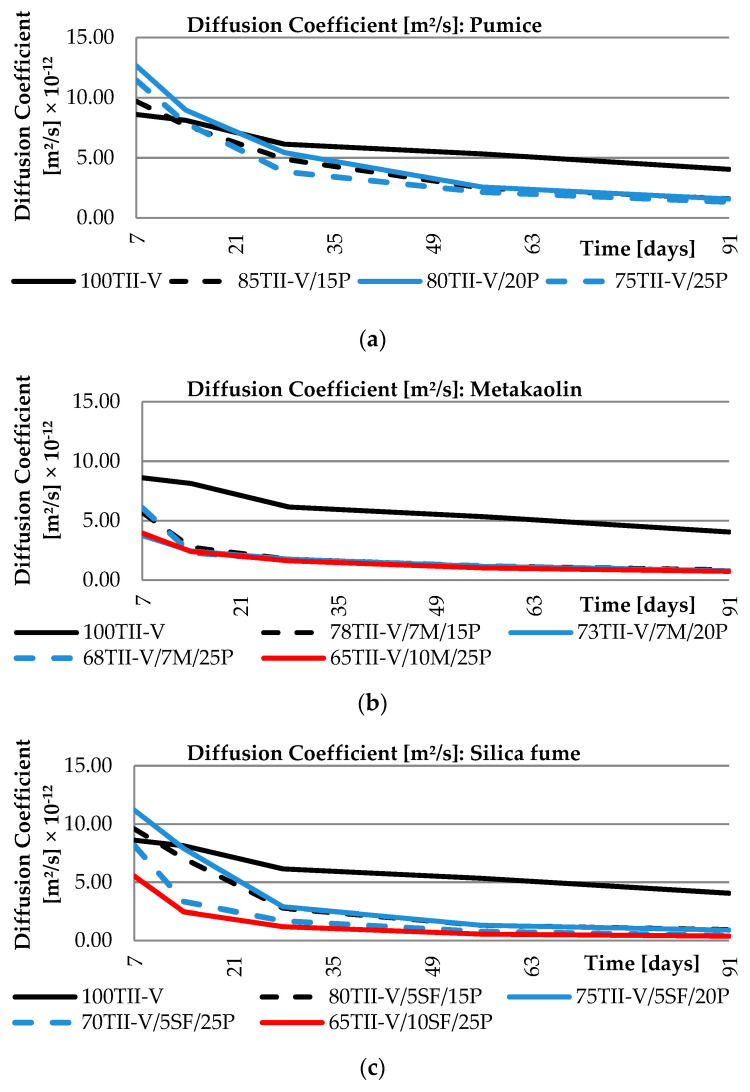
Time-dependent variation of diffusion coefficient: (**a**) pumice group; (**b**) metakaolin group; (**c**) silica fume group.

**Figure 6:**
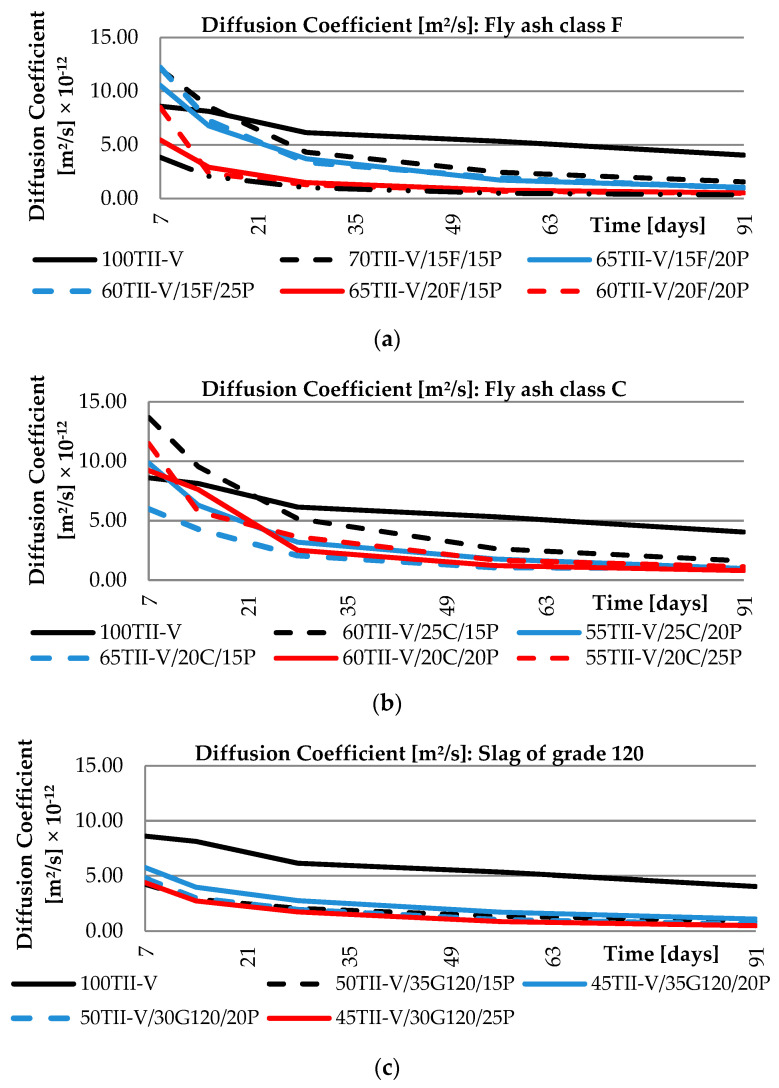
Time-dependent variation of diffusion coefficient: (**a**) fly ash class F group; (**b**) fly ash class C group; (**c**) slag of grade 120 group.

All mixtures were divided into various groups based on typical cementitious material components. The first group is composed of binary mixtures with only pumice substitution. The other group represents ternary mixtures where the pumice is combined with other cementitious materials namely metakaolin, silica fume, fly ash class F, fly ash class C, and ground granulated blast furnace slag class 120. The results of the diffusion coefficients for these groups are shown in Figure 5 and Figure 6.

When evaluating the curves, it can be observed that the diffusion coefficients of binary mixtures with pumice and ternary mixtures with metakaolin are very similar regardless of the amount of replacement of SCM. In the binary-based pumice mixtures, the initial value of the diffusion parameter (ranging from 9.7 × 10^−12^ to 13 × 10^−12^) is higher than the reference concrete (8.6 × 10^−12^) and it decreases over time as the concrete matures, where the pumice mixture ranges (ranging from 13.3 × 10^−12^ to 16.0 × 10^−12^) outperformed the OPC (4.1 × 10^−12^). In the case of ternary mixtures with metakaolin, even the initial value of the diffusion coefficient (ranging from 3.7 × 10^−12^ to 6.1 × 10^−12^) is lower compared to reference OPC concrete, and thus offers higher resistance from the early stage of serviceability. The last scatter of the diffusion coefficient for the metakaolin and SCM with pumice ranges from 0.7 × 10^−12^ to 0.8 × 10^−12^. Mixtures with silica fume shows a larger variance of values on the curve. The initial values range from 5.5 × 10^−12^ to 11.2 × 10^−12^, while the last available data points at the age of 91 days range between 0.3 × 10^−12^ to 0.9 × 10^−12^.

It can be observed from the groups of mixtures with fly ash Class F and C that there is a significant effect of the amount of SCM replacement on the values of diffusion coefficients and there is also a very high value of diffusion coefficients at early ages of hardening. Conversely, for a mixture with slag of grade class 120, the effect on the computed values is not significant. This group of mixtures behaves similarly to the group with metakaolin (initial ranging 4.3 × 10^−12^ to 5.8 × 10^−12^ and the last range is 0.5 × 10^−12^ to 1.1 × 10^−12^). A global view of all VPP mixtures for the calculated diffusion coefficients shows high resistance for chloride ion ingress, as the diffusion coefficient of binary and ternary blends is lower than the reference OPC mixture. However, some pumice, fly ash, and silica fume-based mixtures had a diffusion coefficient higher at initial age (up to 28 days) as the hydration/pozzolanic reaction takes a longer time to complete. Comparing the results of the groups showed that the best performance in early reading showed the group of Metakaolin and Slag of Grade 120, while the group of Fly ash Class F performed the best at the age of 91 days. Since the long-term performance is important with respect to durability, 91-day values are important in order to conclude.

## 5. Coefficient of Variation Analysis

Due to the observed change in the random variation of the diffusion coefficient over time, it is necessary to determine a suitable parameter related to the variance of the measured data for time-dependent probabilistic calculation. A coefficient of variation was chosen similarly as in [4], as it is dimensionless and could allow a suitable description of input parameters. However, it is necessary to evaluate whether the variation coefficient of the diffusion parameter may be considered as constant over time or it can be considered as a time-dependent parameter. In other words, it is important to consider when the mean coefficient of variation might be more appropriate or when the coefficient described with the linear regressions might be used. This approach enables us to understand and describe if the variation coefficient is constant, increasing, or decreasing over time.

### 5.1. Mean Model

Using the error estimator for mean value and measured values of the coefficient of variation was the first approach. The sum of squares expressing the degree of accuracy was computed as follows:

(3) $${\mathrm{SSE}}_{mean}=\sum_{i=0}^{5}{\left[cv_{mean}-cv_{t,\exp}\right]}^{2}$$

where cv*_mean_* is the mean value of the coefficient of variation and *cv_t,exp_* is the coefficient of variation obtained from experimental measurements (see Table 2). To express the accuracy of the method describing random variance, the calculated sum of least square was modified to the root of mean squared error (RMSE):

(4) $${\mathrm{RMSE}}_{mean}=\sqrt{\frac{{\mathrm{SSE}}_{mean}}{n}}$$

where n is equal to 5 and represents the number of measurements over time. The relative root means square error (RRMSE) is computed next to evaluate the significance of the error with respect to the mean value of the diffusion coefficient variation.

(5) $${\mathrm{RRMSE}}_{mean}=\frac{{\mathrm{RMSE}}_{mean}}{cv_{mean}}$$

### 5.2. Trend Line Model

The trend line for the linear regression of the variation of the diffusion coefficient can be expressed as follows:

(6) $$cv_{t,LR}=a-b\times t.\;$$

where *t* is the age in (days), *a* is the characteristics constant, which provides the theoretical variation coefficient at age *t* = 0 days, and *b* is the slope of the trend line. The resulting variation coefficient computed according to the regression via trend line for the age *t* was included in the second calculation of the sum of squares:

(7) $${\mathrm{SSE}}_{LR}=\overset{5}{\underset{i=0}{\sum }}{\left[cv_{t,LR}-cv_{t,\exp}\right]}^{2}$$

The root of mean squared error (RMSE) was also utilized in the second approach:

(8) $${\mathrm{RMSE}}_{LR}=\sqrt{\frac{{\mathrm{SSE}}_{LR}}{n}}.\;$$

Again, the relative root means square error (RRMSE) is computed in the context of the mean value of the diffusion coefficient variation.

(9) $${\mathrm{RRMSE}}_{LR}=\sqrt{\frac{{\mathrm{RMSE}}_{LR}}{{\mathrm{cv}}_{mean}}}$$

### 5.3. Statistical Evaluation

**Figure 7:**
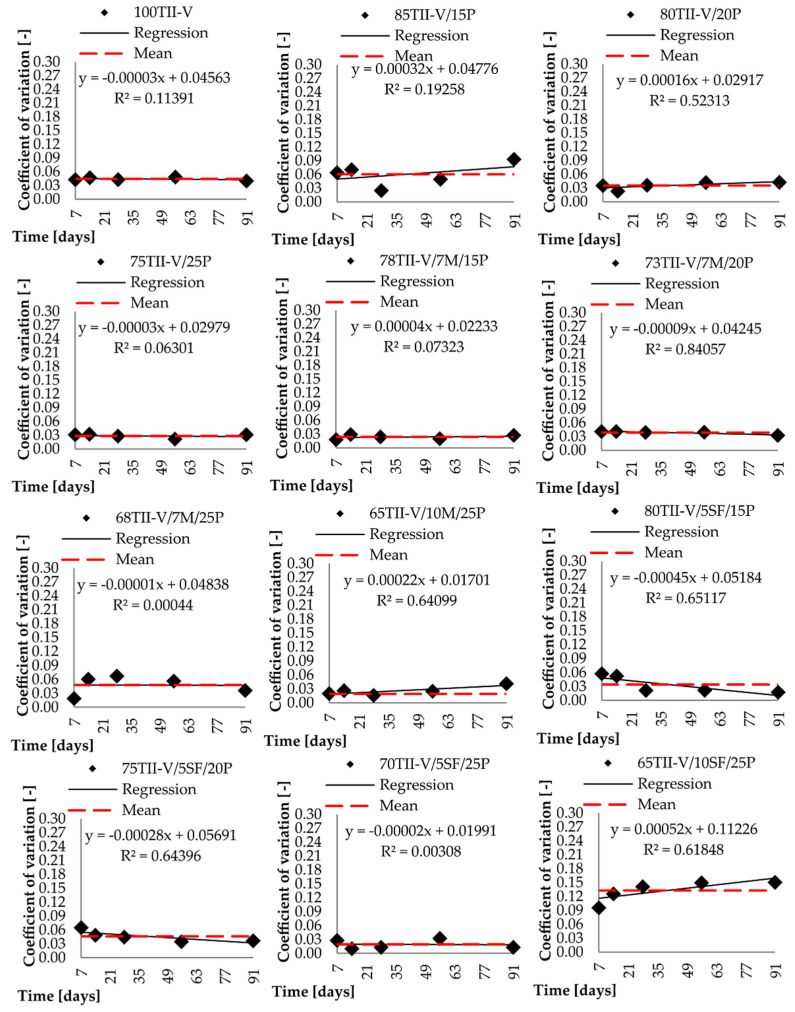
Comparison of experimental data with mean and linear regression approximations of variation coefficients for mixtures groups with pumice, metakaolin, and silica fume.

**Figure 8:**
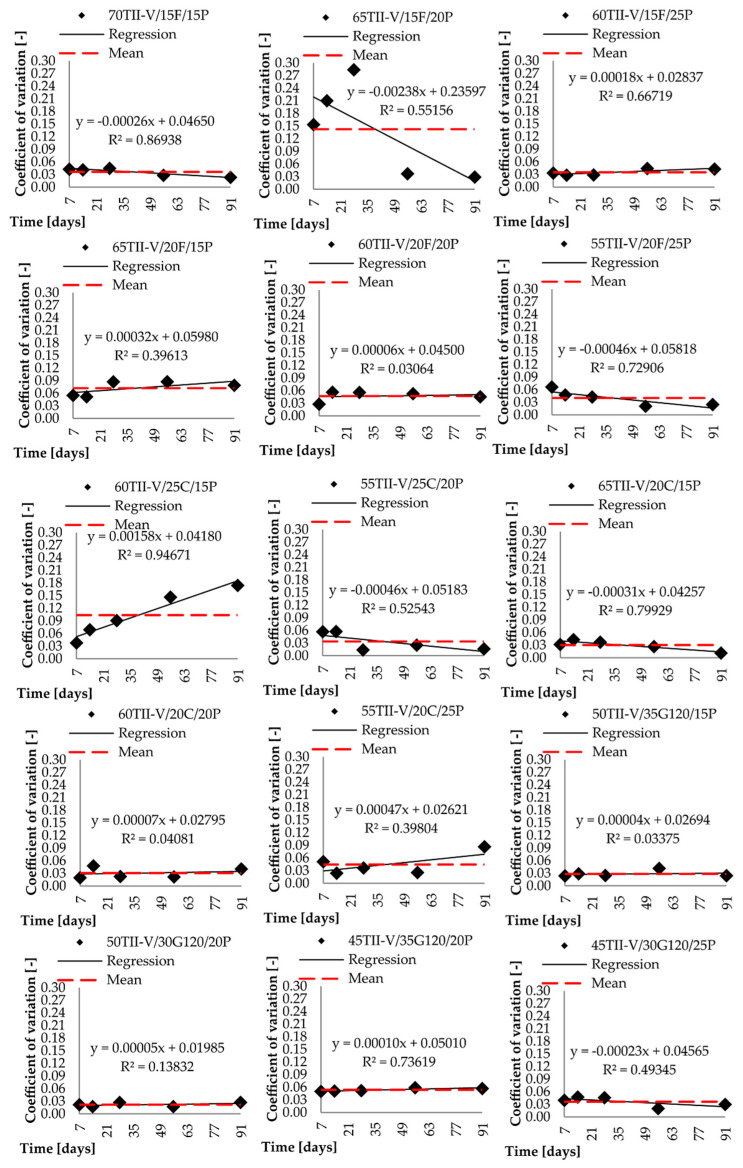
Comparison of experimental data with mean and linear regression approximations of variation coefficients for mixtures groups with fly ash class F, fly ash class C, and slag of grade 120.

Both presented procedures were applied to the investigated data set. In addition to the diffusion coefficient, the standard deviation at each testing day was determined. The calculated coefficient of variation was analyzed, and the results are graphically displayed for each considered mixture in each group and shown in Figure 7 and Figure 8.

In most situations, the results show accordance between the mean and the trend line due to the relatively constant value of the coefficients of variation over time. Two mixtures: 65TII-V/15F/20P and 60TII-V/25C/15P, show the largest deviation. The figures show large differences in the coefficients of variation at various testing days. The mixture 65TII-V/15F/20P also shows a large graphical difference compared to both methodologies. The mixture 60TII-V/25C/15P has a large increase in value over time and thus a large slope of the trend line. The RMSE calculation was implemented to better describe the accuracy of both approaches. Moreover, the R^2^ approach is used as a complementary verification check. It is interesting that the change of *c_v_* over time was highly significant in selected fly ash-based pumice mixtures.

It can be observed that the regression fit described by R^2^, where RMSE and RRMSE favorited approximation of *c*_v_ with mean value, was very low as the dataset showed almost constant behaviour. When the linear regression is favorited and R^2^ is low, as in the cases of the mixtures 65TII-V/15F/20P and 55TII-V/20C/25P, which are 0.552 and 0.398 respectively, this indicates a possible inconsistency in laboratory measurements. Therefore, the conclusions with respect to the suitable approximation approach for the time-dependent variation of the diffusion coefficient shall be considered indicative only. Moreover, the recommendation of 78TII-V/7M/15P for the linear regression with very low R^2^ of 0.073 is the mean value. Therefore, LR recommendation is considered unreliable herein as well.

## 6. RMSE Analysis

**Figure 9:**
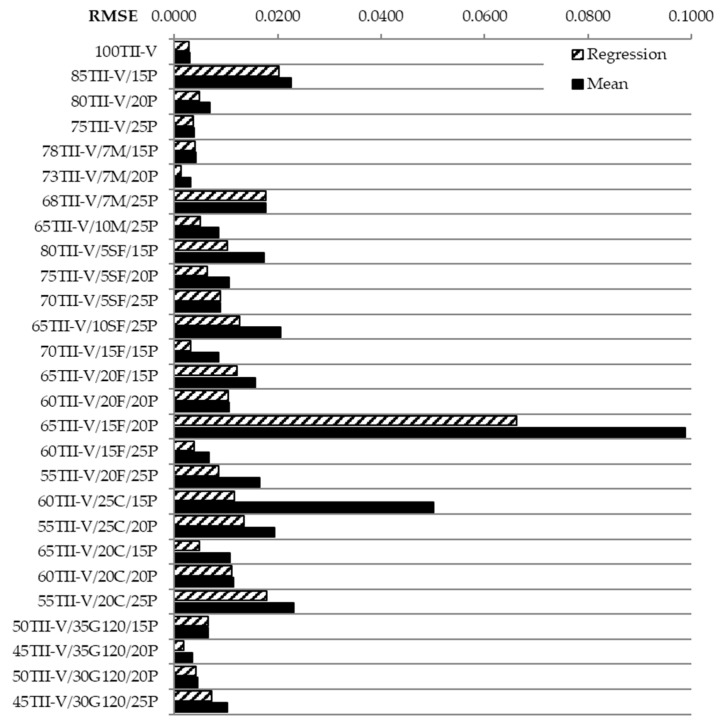
Root mean square error for mean value and trend-line of variation coefficient.

As an example, RMSE calculation is illustrated in the mixture 85TII-V/15P. According to Equation (2), the sum of the squares was calculated as SSE*_mean_* = 2.55 × 10^−3^. Subsequently, according to Equation (3), the SSE*_mean_* was divided by five (number of measurements in time), yielding RMSE*_mean_* = 0.0226. Similarly, according to Equation (6), SSE*_LR_* = 2.06 × 10^−3^ was calculated and according to Equation (7), RMSE*_LR_* = 0.0203. Figure 9 shows RMSE for mean value and trend-line of the variation coefficient. The higher RMSE is because the deviation of approximation from the measured values is higher.

**Figure 10:**
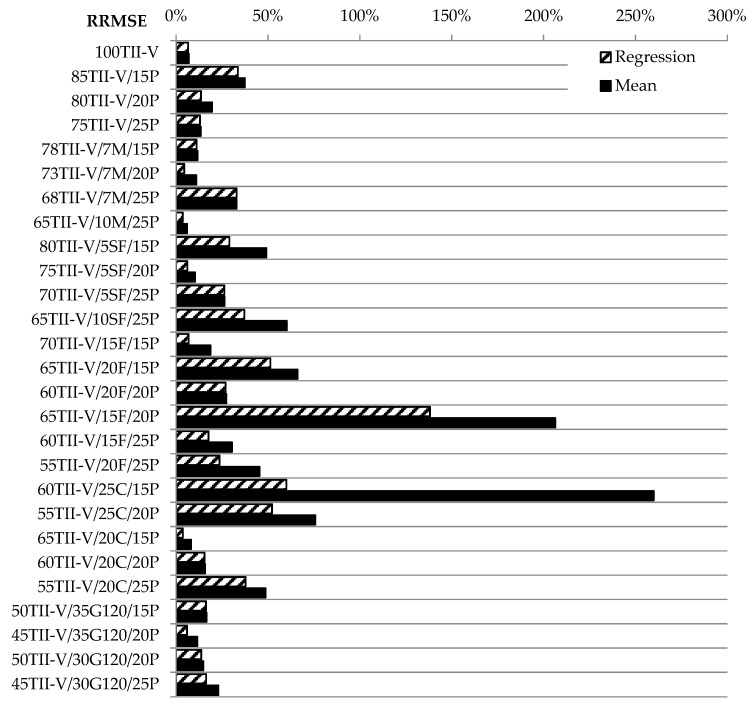
The relative error of the approximation RRMSE of the mean and regression.

The most significant deviation is observed for the mixture 65TII-V/15F/20P for both approximation methods. However, this is most likely attributed to inconsistencies in measurements as indicated by R^2^ Lower values. One should notice the large distance between the measured points by observing Figure 8. Even both approximations do not have the same precision level for this mixture. Very low values and better fit of experimental data with approximation were observed for the reference mixture and other mixtures namely 73TII-V/7M/20P, 45TII-V/35G120/20P, and 70TII-V/15F/15P. It was expected that the linear regression would have better or the same accuracy as the mean-based approach. This was confirmed practically for all concrete mixtures. Another factor that needs to be evaluated is the difference in feasibility between the two approaches. The last phase of statistical analysis is the evaluation of the relative quadratic deviation according to Equations (4) and (8). As an example, a mixture 85TII-V/15P was chosen, where according to Equation (4), the value RMSE*_mean_* = 0.0226 was divided by the value cv*_mean_* = 0.0603 with the result RRMSE*_mean_* = 0.37. Similarly, according to Equation (8), RMSE*_LR_* = 0.0203 was again divided by the value of cv*_mean_* with the result RRMSE*_LR_* = 0.34. Figure 10 shows the calculated values in percentages for both approaches.

The higher the difference between these two approaches is, the worse is the fit by average variation coefficient. There is a large difference between the accuracy of both methods on the mixture 60TII-V/25C/15P. There is also a visible difference for several other mixtures between the two methods (70TII-V/15F/15P, 73TII-V/7M/20P, and 65TII-V/20C/15P), where the difference is higher than 50%. Based on the above mentioned, it is not suggested to use the approximation with the mean value for these mixtures. It should be noted that relations between the amount of SCM and the resulting variance of the data are not observed. It can be seen that the RRMSE values for mixtures 60TII-V/25C/15P and 65TII-V/20C/15P are very high. The difference is more than 100% comparing the RMSE to the mean value of the coefficient of variation, especially for the mean approach. It seems that the difference in the case of RRMSE for more than 75% provides information about the quality of the original data since these are scattered with outliers causing the error. Based on the comparison of regression approach and mean approach, it can be found that the difference between the RRMSE is more than 10% for the following mixtures: 80TII-V/5SF/15P, 65TII-V/10SF/25P, 60TII-V/25C/15P, 55TII-V/20F/25P, 60TII-V/25C/15P, and 55TII-V/20C/25P. However, the conclusions for the mixture 60TII-V/25C/15P are not reliable due to concerns about the measured data.

## 7. Diffusion Coefficient Time-Dependent Variation Model

Based on the RRMSE results, the recommendations for the diffusion coefficient variation in time are presented in Table 3. A suitable model is selected if the ∆RRMSE, which is RRMSE*_mean_*—RRMSE*_LR_*, is higher than 10%. It would be optimal to have also the full time-dependent model for the diffusion coefficient *D*_c,*t*_ itself to be able to have the full set of parameters for the time-dependent ageing model of *D*_c_. However, such approximation available e.g., in [38], would require proper evaluation and approximation that is beyond the scope of this paper.

## 8. Discussion

### 8.1. Diffusion Coefficient

The deterministic diffusion coefficient model data are provided in Table 2 including the reference age of 28 days. However, the study [37] suggests that it needs to be evaluated if *t*_ref_ 56 or 91 would not yield a better fit for the model. The deterministic diffusion coefficient model can be used as the nominal (mean) value for the probabilistic description. The probabilistic analysis also requires a description of the random dispersion. Since the nominal value decreases with ageing, the dimensionless parameter (coefficient of variation) is used [4]. However, the question studied herein is if the variation is constant over the ageing or changes linearly. Differences between those two approaches favoring linear regression were observed for several concrete mixtures blended with fly ash admixture as shown in the results section. The suitable approximation models for each mixture (mean or linear regression) are indicated as the diffusion coefficient time-dependent variation model analysis. The RMSE analysis summarized in Table 3 reveals that seven mixtures including the reference one can be described by the mean value of the coefficient of variation of the diffusion coefficient parameter. The second approach based on the linear regression of coefficient of variation over the concrete age proves to be justified for 10 mixtures. For the other 10 mixtures, the recommendation of the selection of methods was considered unreliable. It should be noted that the variance of the input values may be also caused by the accuracy of the test methods and no suitable correlation was found in between the amount of SCMs and the variance of the data from the results of the examined concrete mixture samples. Since it is reasonable to adopt the linear regression approach, it is very important to study the performance of both types of statistical descriptions and their effect on the actual distribution of chloride concentration at the reinforcement level in the typical cases. Therefore, the numerical study of chloride ingress into concrete that would evaluate both studied models (mean approximation and linear regression one) would reveal new insight into the actual effect of the model selection. The differences in actual simulated concentration profiles would give a more relevant answer to practical necessity distinguished between both approaches.

### 8.2. Compressive Strength

It could be observed that 21 mixtures have an *f*_c,56_/*f*_c,28_ ratio higher than 110% and 10 mixtures have an *f*_c,91_/*f*_c,2_ ratio higher than 130%. Therefore, it can be seen that the 28th day strength is not a proper description of those mixtures with respect to the design process. The lowest value of *f*_c,91_/*f*_c,28_ = 104% has the concrete mixture 78TII-V/7M/15P. It is worth mentioning that these results seem more like the error since the *f*_c,56_/*f*_c,28_ is 113%, which would imply that the strength is decreasing over time. It would be valuable to study also the time-dependent analysis of variation of compressive strength. However, it is not performed since there are not enough data points allowing to compute scatter of compressive strength over time.

## 9. Conclusions

This study demonstrates the statistical description of the variability of the chloride diffusion coefficient with volcanic pumice-based SCM. The improved methodology for the statistical evaluation of time-dependent behaviour of the variation of the diffusion coefficient for binary and ternary mixtures was applied. In addition, the deterministic time-dependent description of the compressive strength is provided to provide a complementary input data set with respect to Ultimate Limit State (ULS) analysis.

These conclusions were drawn from statistical analysis of OPC mixture and 26 various binary and ternary concrete mixtures containing VPP:

1. Based on the RRMSE results, seven mixtures are recommended for the mean value of variation coefficient, and 10 mixtures are recommended for the linear regression of variation coefficient (see Table 3).
2. For the other 10 mixtures, the recommendation of the selection of methods was considered unreliable.
3. The diffusion coefficient results of Class F and Class C-based mixtures show that there is a significant effect of the amount of SCM on the values of diffusion coefficients, and there is also a very high value of diffusion coefficients at early ages of hardening.
4. Analysis results of the time parameter of compressive strength have confirmed that 56 days and 91 days are more appropriate compared to 28 days strength.
5. The comparison of the results between the groups of binary and ternary mixtures showed that the best performance for the matured concrete (at the age of 91 days) was observed in the group of Class F-based mixtures blended with pumice.
6. In summary, this research will lead a pathway for the practical application of pumice materials in future bridge deck slabs based on their effectiveness of replacement and interaction with other cementitious materials.

## Figures and Tables

**Table 1:** Concrete mixtures’ cementitious material constituents and proportions [%].

Mix ID	Cement	Pumice	Fly Ash C	Fly Ash F	Slag G120	Silica Fume	Metakaolin
100TII-V	100	–	–	–	–	–	–
85TII-V/15P	85	15	–	–	–	–	–
80TII-V/20P	80	20	–	–	–	–	–
75TII-V/25P	75	25	–	–	–	–	–
78TII-V/7M/15P	78	15	–	–	–	–	7
73TII-V/7M/20P	73	20	–	–	–	–	7
68TII-V/7M/25P	68	25	–	–	–	–	7
65TII-V/10M/25P	65	25	–	–	–	–	10
80TII-V/5SF/15P	80	15	–	–	–	5	–
75TII-V/5SF/20P	75	20	–	–	–	5	–
70TII-V/5SF/25P	70	25	–	–	–	5	–
65TII-V/10SF/25P	65	25	–	–	–	10	–
70TII-V/15F/15P	70	15	–	15	–	–	–
65TII-V/15F/20P	65	20	–	15	–	–	–
60TII-V/15F/25P	60	25	–	15	–	–	–
65TII-V/20F/15P	65	15	–	20	–	–	–
60TII-V/20F/20P	60	20	–	20	–	–	–
55TII-V/20F/25P	55	25	–	20	–	–	–
60TII-V/25C/15P	60	15	25	–	–	–	–
55TII-V/25C/20P	55	20	25	–	–	–	–
65TII-V/20C/15P	65	15	20	–	–	–	–
60TII-V/20C/20P	60	20	20	–	–	–	–
55TII-V/20C/25P	55	25	20	–	–	–	–
50TII-V/30G120/20P	50	20	–	–	30	–	–
50TII-V/35G120/15P	50	15	–	–	35	–	–
45TII-V/35G120/20P	45	20	–	–	35	–	–
45TII-V/30G120/25P	45	25	–	–	30	–	–

**Table 2:** Mean value of diffusion coefficient *µ* (m^2^/s) and variation coefficient *c_v_* (-) in time *t* (days), and calculated aging factor *m* (-) and compressive strength *f_c,28_* (MPa).

No.	*µ* (m^2^/s) ×10^−12^	*c_v_* (-)	*µ* (m^2^/s) ×10^−12^	*c_v_* (-)	*µ* (m^2^/s) ×10^−12^	*c_v_* (-)	*µ* (m^2^/s) ×10^−12^	*c_v_* (-)	*µ* (m^2^/s) ×10^−12^	*c_v_* (-)	*m* (-)	*f_c,28_* (MPa)
Time (Days)	7		14		28		56		91			
100TII-V	8.61	0.043	8.12	0.047	6.14	0.043	5.33	0.049	4.05	0.040	0.264	27.98
85TII-V/15P	9.71	0.064	7.68	0.071	4.90	0.025	2.49	0.049	1.60	0.093	0.521	39.57
80TII-V/20P	1.27	0.035	8.97	0.229	5.44	0.036	2.57	0.042	1.58	0.042	0.628	34.97
75TII-V/25P	1.15	0.031	7.88	0.032	3.85	0.028	2.15	0.021	1.33	0.031	0.807	35.44
78TII-V/7M/15P	5.69	0.018	2.76	0.029	1.77	0.024	1.18	0.020	8.69	0.027	0.764	31.60
73TII-V/7M/20P	3.75	0.041	2.45	0.041	1.77	0.039	1.16	0.040	7.95	0.033	0.522	37.43
68TII-V/7M/25P	6.08	0.019	2.29	0.060	1.71	0.067	1.19	0.056	7.61	0.036	0.532	34.92
65TII-V/10M/25P	3.96	0.020	2.40	0.026	1.62	0.016	1.02	0.025	7.32	0.041	0.766	39.35
80TII-V/5SF/15P	9.59	0.058	7.04	0.053	2.76	0.021	1.30	0.022	9.50	0.017	0.936	28.26
75TII-V/5SF/20P	1.12	0.065	7.89	0.049	2.89	0.045	1.31	0.035	9.06	0.037	0.721	37.81
70TII-V/5SF/25P	8.18	0.028	3.34	0.010	1.69	0.013	7.86	0.032	4.40	0.013	0.825	35.16
65TII-V/10SF/25P	5.53	0.096	2.46	0.126	1.20	0.141	5.57	0.150	3.82	0.151	0.932	32.31
70TII-V/15F/15P	1.21	0.043	8.57	0.042	4.32	0.045	2.45	0.029	1.56	0.023	1.000	32.40
65TII-V/15F/20P	1.06	0.154	6.77	0.210	3.72	0.284	1.71	0.037	1.04	0.029	0.826	48.65
60TII-V/15F/25P	1.22	0.033	7.28	0.028	3.39	0.029	1.92	0.044	9.83	0.043	0.537	48.72
65TII-V/20F/15P	5.46	0.055	2.91	0.052	1.49	0.088	7.80	0.088	5.26	0.079	0.883	49.11
60TII-V/20F/20P	8.51	0.028	2.35	0.056	1.28	0.055	6.89	0.053	4.82	0.045	0.651	44.05
55TII-V/20F/25P	3.84	0.066	2.08	0.047	1.01	0.042	4.86	0.020	3.32	0.025	0.675	42.50
60TII-V/25C/15P	1.37	0.038	9.55	0.069	5.17	0.091	2.64	0.147	1.62	0.174	1.000	45.66
55TII-V/25C/20P	9.85	0.057	6.31	0.058	3.20	0.014	1.78	0.025	9.87	0.016	0.636	45.24
65TII-V/20C/15P	5.99	0.032	4.29	0.043	2.07	0.037	1.06	0.027	1.01	0.012	1.000	41.16
60TII-V/20C/20P	9.21	0.020	7.63	0.048	2.52	0.023	1.23	0.022	8.06	0.041	0.937	31.12
55TII-V/20C/25P	1.15	0.051	5.76	0.024	3.65	0.036	1.68	0.025	1.13	0.087	1.000	32.00
50TII-V/30G120/20P	4.79	0.022	2.99	0.017	1.95	0.027	1.00	0.017	6.06	0.027	0.968	32.02
50TII-V/35G120/15P	4.25	0.024	2.89	0.028	2.06	0.025	1.33	0.041	9.22	0.024	0.793	38.96
45TII-V/35G120/20P	5.75	0.050	3.99	0.051	2.77	0.052	1.71	0.059	1.08	0.057	0.986	46.06
45TII-V/30G120/25P	4.40	0.040	2.73	0.047	1.73	0.046	8.55	0.020	4.81	0.030	0.818	46.12

**Table 3:** Recommendation for the time-dependent diffusion coefficient model mean value *c_v,mean_* and linear regression *c_v.LR_*.

No.	Mean		Linear Regression			∆RRMSE	Recommen.
	*c_v.mean_* (-)	RRMSE*_mean_*	*c_v,LR_* (-)	R^2^*_LR_*	RRMSE*_LR_*		
100TII-V	0.0443	7%	−0.00003x + 0.04563	0.114	7%	0%	Mean
85TII-V/15P	0.0603	37%	0.00032x + 0.04776	0.193	34%	4%	LR
80TII-V/20P	0.0356	20%	0.00016x + 0.02917	0.523	14%	6%	LR
75TII-V/25P	0.0286	13%	−0.00003x + 0.02979	0.063	13%	0%	Mean
78TII-V/7M/15P	0.0238	12%	0.00004x + 0.02233	0.073	11%	0%	Mean
73TII-V/7M/20P	0.0388	11%	−0.00009x + 0.04245	0.841	4%	7%	LR
68TII-V/7M/25P	0.0479	33%	−0.00001x + 0.04838	0.000	33%	0%	Mean
65TII-V/10M/25P	0.0257	6%	0.00022x + 0.01701	0.641	4%	2%	LR
80TII-V/5SF/15P	0.0341	49%	−0.00045x + 0.05184	0.651	29%	20%	Not reliable
75TII-V/5SF/20P	0.0460	10%	−0.00028x + 0.05691	0.644	6%	4%	LR
70TII-V/5SF/25P	0.0193	26%	−0.00002x + 0.01991	0.003	26%	0%	Mean
65TII-V/10SF/25P	0.1328	60%	0.00052x + 0.11226	0.618	37%	23%	Not reliable
70TII-V/15F/15P	0.0363	19%	−0.00026x + 0.04650	0.869	7%	12%	Not reliable
65TII-V/15F/20P	0.1427	206%	−0.00238x + 0.23597	0.552	138%	68%	Not reliable
60TII-V/15F/25P	0.0353	31%	0.00018x + 0.02837	0.667	18%	13%	Not reliable
65TII-V/20F/15P	0.0724	66%	0.00032x + 0.05980	0.396	51%	15%	Not reliable
60TII-V/20F/20P	0.0474	27%	0.00006x + 0.04500	0.031	27%	0%	Mean
55TII-V/20F/25P	0.0402	45%	−0.00046x + 0.05818	0.729	24%	22%	Not reliable
60TII-V/25C/15P	0.1037	260%	0.00158x + 0.04180	0.947	60%	200%	Not reliable
55TII-V/25C/20P	0.0339	76%	−0.00046x + 0.05183	0.525	52%	24%	Not reliable
65TII-V/20C/15P	0.0303	8%	−0.00031x + 0.04257	0.799	4%	5%	LR
60TII-V/20C/20P	0.0309	16%	0.00007x + 0.02795	0.041	15%	0%	Mean
55TII-V/20C/25P	0.0447	49%	0.00047x + 0.02621	0.398	38%	11%	Not reliable
50TII-V/30G120/20P	0.0220	15%	0.00005x + 0.01985	0.138	14%	1%	LR
50TII-V/35G120/15P	0.0285	17%	0.00004x + 0.02694	0.034	16%	0%	Mean
45TII-V/35G120/20P	0.0539	12%	0.00010x + 0.05010	0.736	6%	6%	LR
45TII-V/30G120/25P	0.0365	23%	−0.00023x + 0.04565	0.493	16%	7%	LR

## Data Availability

Data is contained within the article.
